# COVID-19 mortality risk among people with HIV in Florida before and after the introduction of COVID-19 vaccines: A population-based study

**DOI:** 10.1371/journal.pone.0358543

**Published:** 2026-09-18

**Authors:** Tendai N.R. Gwanzura, Mary Jo Trepka, Tan Li, Levente Juhasz, Giselle A. Barreto, Shelbie Burchfield, Diana M. Sheehan

**Affiliations:** 1 Robert Stempel School of Public Health and Social Work, Department of Epidemiology, Florida International University, Miami, Florida, United States of America; 2 Robert Stempel School of Public Health and Social Work, Department of Biostatistics, Florida International University, Miami, Florida, United States of America; 3 Ft. Lauderdale Research & Education Center, School of Forest, Fisheries and Geomatics Sciences, Geospatial Analytics, Technology, and Open Research (GATOR) Lab, University of Florida, Davie, Florida, United States of America; Taipei Medical University, TAIWAN

## Abstract

**Objective:**

Assess associations between COVID-19 vaccine availability and differences in COVID-19 mortality risk across subgroups among 122,527 people with HIV (PWH) in Florida.

**Methods:**

Analyzed HIV surveillance data, Social Vulnerability Index (SVI), and Rural-Urban Commuting Area Codes using a competing risks model to compare COVID-19 mortality among PWH before (3/1/2020–4/30/2021) and during (5/1/2021–12/31/2021) vaccine availability.

**Results:**

Overall COVID-19 mortality rates increased from before to during the vaccine availability period (22.3 vs. 27.5 per 100,000 person-months), driven in part by the Delta variant surge in mid-to-late 2021. Compared to Non-Hispanic White (NHW) PWH, adjusted COVID-19 mortality hazards remained elevated for Non-Hispanic Black (NHB) PWH both before (HR 2.06, 95% CI 1.45–2.91) and during (HR 1.60, 95% CI 1.13–2.27) vaccine availability. For Hispanic PWH, the relative hazard compared to NHW was elevated before (HR 2.24, 95% CI 1.55–3.23) and attenuated during vaccine availability (HR 1.03, 95% CI 0.68–1.57). In the final model, living in ZIP Code-level areas of high social vulnerability (overall SVI high vs. low) was associated with elevated hazard during vaccine availability (HR 1.75, 95% CI 1.04–2.97).

**Conclusions:**

Overall COVID-19 mortality rates were higher during the vaccine availability period (27.5 vs. 22.3 per 100,000 person-months); however, subgroup patterns varied substantially. Inequities persisted in key subgroups and were most pronounced among PWH living in more socially vulnerable areas. These observational comparisons do not estimate the causal effect of vaccination uptake.

**Public health implications:**

Tailored vaccination strategies and structural interventions remain essential to advancing equity in pandemic response among PWH.

## Introduction

COVID-19 emerged as the third leading cause of death in the United States (US) between 2020 and 2021, with 767,724 fatalities [1–3]. Studies indicate mixed results on COVID-19 mortality among people with HIV (PWH). One meta-analysis found higher mortality among PWH than among people without HIV [4], while another found no statistically significant increase in COVID-19 mortality associated with HIV [5]. A pooled analysis conducted as part of a scoping review identified COVID-19 mortality differences among PWH related to age, sex, race, injection drug use, comorbidities, smoking, and CD4 count [6]. Specifically, those aged 70 years or older had a higher case fatality rate of 41.8% compared to 1.2% among those under 40 years of age [6]. Additionally, the pooled case fatality rate for males was approximately twice that of females [6]. Notably, Black PWH had a higher case fatality rate (12.9%), than Hispanics (10.3%) and White PWH (10.0%) [6]. Those reporting injection drug use, smoking, and having a CD4 cell count below 200 had higher pooled case-fatality rates [6]. A study of US counties found that mortality risk was also higher in counties with a higher percentage of the Black population, in both rural and urban settings [7].

Although few studies have examined COVID-19 outcomes among PWH in the context of community vulnerability, one study in Florida’s general population found that counties with a high Social Vulnerability Index (SVI) had a 19.4% higher COVID-19 mortality rate than the national average [8]. This suggests that structural factors—like poverty, crowding, and transportation access—may also shape mortality patterns among PWH.

The advent and widespread distribution of COVID-19 vaccines represented a critical milestone in mitigating mortality rates [9,10]. Notable differences in COVID-19 mortality persisted after vaccine introduction, with disparities particularly pronounced among racial/ethnic minorities and older adults [3,11]. In a community-based Connecticut sample surveyed before vaccine availability, vaccine hesitancy was identified as a contributing factor, with higher hesitancy observed among Black and Hispanic communities compared to non-Hispanic White/other communities [12]. Counties with 80% or more vaccination coverage saw 46% lower COVID-19 mortality rates compared to those with less than 50% coverage [13]. Additionally, counties with 70–79% and those with 60–69% vaccination rates had a 35% and 20% reduction in mortality rates, respectively [13].

A statewide spatial analysis in Florida found that vaccination sites were concentrated in urban counties, with rural counties having fewer sites and longer travel times to nearest vaccination sites [14]. Additionally, research conducted in New York showed that 63.5% of PWH had completed a primary COVID-19 vaccination regimen compared to 75.0% in the general population [15]. These differences suggest that improving vaccine accessibility is key to ensuring equitable health outcomes and reducing mortality.

Despite the extensive research on differences in COVID-19 mortality among the general population, limited data exist on how vaccine availability influenced mortality differences among PWH [6]. To address this gap, we evaluated the effect of COVID-19 vaccine availability on COVID-19 mortality across socio-demographic and community-level factors among PWH in Florida.

## Methods

### Study population and data sources

We conducted a retrospective cohort study of 129,770 PWH residing in Florida, followed from March 1, 2020, to December 31, 2021, using data from the Florida Department of Health’s (FDOH) enhanced HIV/AIDS Reporting System (eHARS). This dataset includes demographics (age, sex, race/ethnicity), place of birth, HIV transmission mode, viral suppression status, vital status, underlying cause of death, and ZIP Code Tabulation Areas (ZCTAs) for residence during life and at death. ZCTAs are generalized representations of US postal ZIP Codes created by the US Census Bureau for statistical purposes, approximating the geographic areas covered by ZIP Codes but aligned with census boundaries [16]. Vital status and cause of death were verified through routine linkages to FDOH Vital Records, the Social Security Administration’s Death Master File, and the National Death Index. We used a deidentified dataset linked in June 2022.

### Inclusion and exclusion criteria

Of the 129,770 PWH in the dataset, 122,527 (94.4%) individuals met the inclusion criteria. We included PWH aged 18 years or older residing in Florida by December 31, 2019, and alive during the study period or until death. We excluded 131 (0.1%) individuals over the age of 90 due to potential data quality issues such as records misclassifying deceased individuals. After linking community-level social vulnerability data, 4,340 individuals were removed due to lack of valid or missing ZCTA. Among those who died during the study period, we additionally excluded 2,682 individuals who either died outside Florida or lacked a valid ZCTA at the time of death, including three COVID-19 deaths with no recorded ZCTA or state of residence at death. For PWH missing a ZCTA at the end of 2021 but recorded as alive and residing in Florida, their 2020 ZCTA was used. Of the 122,527 individuals in the final analytic cohort, 3,359 (2.7%) had an unknown place of birth and were retained in the descriptive analyses but excluded from multivariable regression models due to inability to classify US birth status; the final regression model included 119,168 (97.3%) PWH.

### COVID-19 vaccine availability

In the US, the first doses of COVID-19 vaccine were administered on December 14, 2020 [17]. The Advisory Committee on Immunization Practices (ACIP) recommended that adults aged 16–64 years with high-risk medical conditions be offered COVID-19 vaccination during Phase 1c [18]. As vaccine eligibility and supply expanded nationally beginning in late March 2021 [17], the period of vaccine availability for this analysis was defined as starting May 1, 2021, approximately six weeks later. This date coincided with 8.87 million Florida residents having received at least one COVID-19 vaccine dose, including 6.28 million who had completed a vaccine series, as of April 30, 2021, according to the Florida Department of Health COVID-19 Vaccine Summary [19].

### Outcome definition

The primary outcome was COVID-19–related death, classified using International Classification of Diseases 10 (ICD-10 code U07.1). All other deaths were treated as competing events.

### Covariates

Individual and community factors of interest were determined from the literature [5–8]. Individual factors included age, sex, place of birth (US or foreign), and HIV transmission category (injection drug use [IDU]—including men who have sex with men [MSM] who also had the IDU category, MSM, heterosexual transmission, and ‘other’ categories like perinatal transmission, unknown, and blood transfusion). Additional factors included viral suppression status and race/ethnicity (Hispanic, of all races; Non-Hispanic Black [NHB]; Non-Hispanic White [NHW]; and other races including multiple races, Asian, Native American, and Pacific Islander).

Community factors were social vulnerability and rurality at the ZCTA level, and county-level vaccination rates by December 31, 2021. Social vulnerability was assessed at the ZIP Code-level modeling the CDC’s Social Vulnerability Index (SVI), ranking ZCTAs in Florida based on four themes: socioeconomic status, household characteristics, racial/ethnic minority status/language, and housing type/transportation [20]. Because the CDC provides SVI scores at the census tract and county levels but not at the ZCTA level, we replicated the methods for calculating social vulnerability using data from the 2020 5-year American Community Survey (ACS) at the ZCTA level [21]. Each theme was individually scored and combined to create an overall SVI score and theme scores. Subsequently, scores were ranked from 0 to 1 and categorized into tertiles: low (0–0.333), moderate (0.334–0.666), and high (0.667–1), with higher values indicating greater social vulnerability. Rural-urban classifications were determined using Rural-Urban Commuting Area (RUCA) codes [22]. Neighborhood ZCTA-level data were linked with individual-level eHARS data using the ZCTA of residence recorded at death or the most recent ZCTA if the individual did not die.

### Statistical analysis

At-risk time origin: We defined the analytic at-risk period to begin March 1, 2020. Individuals with competing (non-COVID) deaths in January–February 2020 were excluded from the risk set; for all others, time was rebased so that month 0 corresponds to March 2020.

Total person-time, measured in months, was calculated for two groups: before vaccine (deaths from March 1, 2020, to April 30, 2021) and during vaccine availability (deaths from May 1, 2021, to December 31, 2021), from the group start date to the COVID-19 death or end date, with mortality rates calculated as the number of deaths divided by the total person-time in months, multiplied by 100,000. We first used a multilevel cause-specific hazard model to estimate the intraclass correlation at the ZIP Code and county levels, but findings showed minimal clustering. As such, we applied a Fine-Gray proportional sub-distribution hazard model without random effects to estimate the cumulative incidence function (CIF) for COVID-19 deaths, accounting for competing risks among PWH in Florida before and during vaccine availability. Gray’s test was used to assess differences in CIF curves across subgroups. We estimated adjusted sub-distribution hazard ratios (HR) and 95% confidence intervals (CIs) for both periods, controlling for covariates significant in univariate CIF analyses. Statistical significance was set at *P* < 0.05. We conducted analyses in SAS version 9.4 (SAS Institute, Cary, NC).

### Ethics statement

This study was reviewed by the Florida International University Office of Research Integrity and deemed exempt from human subjects review via the Exempt Review process (IRB Protocol Exemption #IRB-22-0517; Exemption Date: 12/08/2022; TOPAZ Reference #113536) and Florida Department of Health (2021-491). The study used de-identified surveillance data provided by the Florida Department of Health, and no informed consent was required.

## Results

The overall COVID-19 mortality rate increased from before to during vaccine availability (22.3 vs. 27.5 deaths per 100,000 person-months), a pattern consistent with the Delta variant surge observed in mid-to-late 2021. Stratified rates revealed demographic and structural differences. Mortality rates among PWH aged 65 and older were similarly elevated before and during vaccine availability (66.0 and 65.6 deaths per 100,000 person-months), whereas rates among those aged 18–34 increased from 4.0 to 8.0 deaths per 100,000 person-months. NHB PWH had the highest mortality rates in both periods, with rates increasing from 28.2 to 37.8 deaths per 100,000 person-months, whereas Hispanics had a notable reduction (21.2 to 16.8 deaths per 100,000 person-months). Heterosexual PWH had the highest increase in rates across transmission categories (30.7 to 40.2 deaths per 100,000 person-months), while those with IDU had similar rates before and during vaccine availability (34.7 and 37.0 deaths per 100,000 person-months, respectively). Rates were similar between PWH with and without viral suppression before vaccine availability (22.2 and 22.4 deaths per 100,000 person-months, respectively), but diverged sharply during vaccine availability, with PWH without viral suppression having a markedly higher rate (38.1 vs. 22.6 deaths per 100,000 person-months). Rural mortality rates decreased (23.2 to 15.2 deaths per 100,000 person-months), while urban rates increased (22.3 to 27.8 deaths per 100,000 person-months). Rates remained highest in ZIP Codes with high social vulnerability during both time periods (25.0 and 30.6 deaths per 100,000 person-months) (Table 1).

**Table 1:** Comparison of COVID-19 mortality rates and person-time among people with HIV before (March 2020 – April 2021) and during vaccine availability (May 2021 – December 2021), Florida.

Characteristic	Before vaccine (March 2020 – April 2021)			During vaccine (May 2021 – December 2021)		
	n	Person time (months)^a^	Rate per 100,000 person-months^b^	n	Person time (months)	Rate per 100,000 person-months
Total population	353	1,471,743	22.3	294	1,001,387	27.5
**Sex**						
Female	104	388,162	26.0	91	263,575	33.8
Male	249	1,083,581	20.9	203	737,812	25.2
**Age at time of death (years)**						
18-34	8	199,565	4.0	11	137,269	8.0
35-49	34	403,457	7.9	39	276,370	13.0
50-64	143	639,788	21.4	135	435,203	29.4
65 and older	168	228,887	66.0	109	152,518	65.6
**Race/ Ethnicity**						
Hispanic	90	381,696	21.2	47	261,531	16.8
Non-Hispanic Black	190	644,269	28.2	174	437,001	37.8
Non-Hispanic White	67	413,939	14.3	69	281,200	22.0
Other	6	31,839	18.8	4	21,655	18.5
**HIV transmission category**						
Heterosexual	151	492,205	30.7	134	333,735	40.2
Injection drug use^c^	49	141,323	34.7	35	94,668	37.0
Men who have sex with men	111	734,777	15.1	94	502,508	18.7
Other	42	103,438	16.4	31	70,476	17.0
**Born in US**						
Yes	269	1,030,274	24.0	228	698,917	30.0
No	81	401,837	19.4	63	275,347	22.5
Unknown^d^	3	39,632	7.6	3	27,123	11.1
**Viral suppression** ^**e**^						
Suppressed	238	994,150	22.2	165	688,892	22.6
Unsuppressed	115	477,593	22.4	129	312,495	38.1
**Rural/urban status**						
Rural	9	38,746	23.2	4	26,301	15.2
Urban	344	1,432,997	22.3	290	975,086	27.8
**Social vulnerability index of ZIP Code at time of death**						
**Overall index** ^**f**^						
Low	26	144,823	15.2	19	98,848	17.2
Medium	62	328,217	17.1	52	223,849	22.3
High	265	998,703	25.0	223	678,690	30.6
**Socioeconomic status (Theme 1 of social vulnerability index)**						
Low	31	215,535	13.5	24	147,280	14.9
Medium	96	424,363	20.0	73	289,334	24.5
High	226	831,845	25.7	197	564,773	32.2
**Household composition and disability (Theme 2 of social vulnerability index)**						
Low	58	315,820	16.8	52	215,647	23.2
Medium	108	493,033	20.5	89	335,865	24.4
High	187	662,890	26.2	153	449,875	31.8
**Minority status and language (Theme 3 of social vulnerability index)**						
Low	36	163,310	19.6	30	110,901	25.2
Medium	83	417,290	19.2	67	283,603	22.9
High	234	891,143	24.2	197	606,883	30.0
**Housing type and transportation (Theme 4 of social vulnerability index)**						
Low	31	130,486	18.4	21	88,927	19.1
Medium	88	458,071	17.9	79	312,206	24.3
High	234	883,186	25.1	194	600,254	30.3

MSM = men who have sex with men. SVI = Social Vulnerability Index.

^a^Person-time = time from cohort entry to COVID-19 death or study end.

^b^The n column reports raw COVID-19 death counts; mortality rates were calculated using weighted COVID-19 deaths and weighted person-time: (weighted COVID-19 deaths ÷ weighted person-time) × 100,000.

^c^Injection drug use includes MSM and heterosexuals who inject drugs.

^d^Individuals with unknown US birth status (n = 3,359; 2.7%) are included in the cohort counts and rates above, but were excluded from multivariable regression analyses; the final regression model included 119,168 (97.3%) PWH.

^e^Viral suppression defined as HIV RNA < 200 copies/mL (most recent test).

^f^SVI tertiles: Low (0–0.333), Medium (0.334–0.666), High (0.667–1).

**Fig 1:**
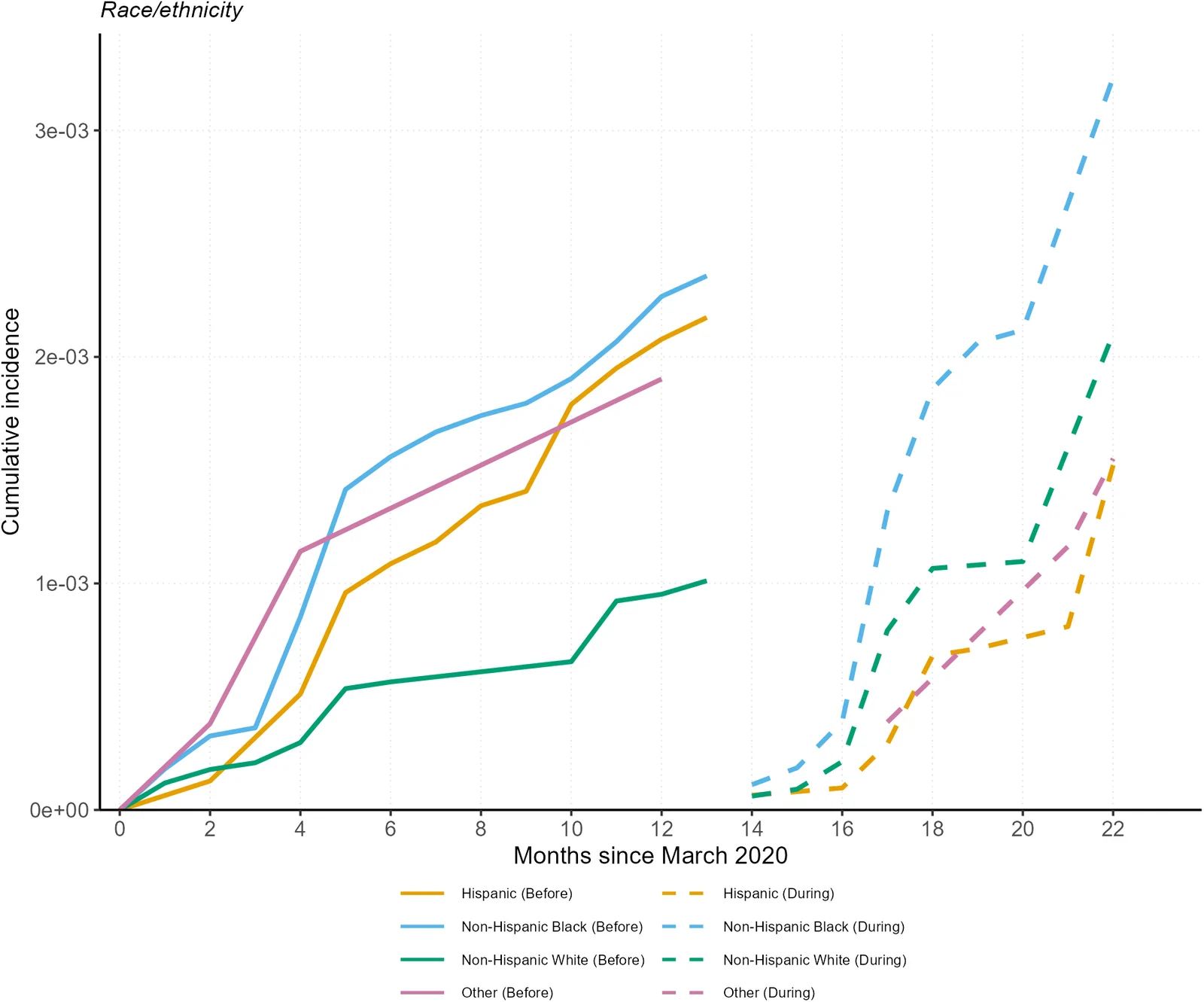
Cumulative incidence of COVID-19 deaths among people with HIV by race/ethnicity before and during vaccine availability, Florida, 2020–2021. Cumulative incidence estimates are shown for Hispanic, non-Hispanic Black (NHB), non-Hispanic White (NHW), and Other race/ethnicity. Solid lines represent the period before vaccine availability (0–13 months since March 2020) and dashed lines represent the period during vaccine availability (14–22 months since March 2020).

**Fig 2:**
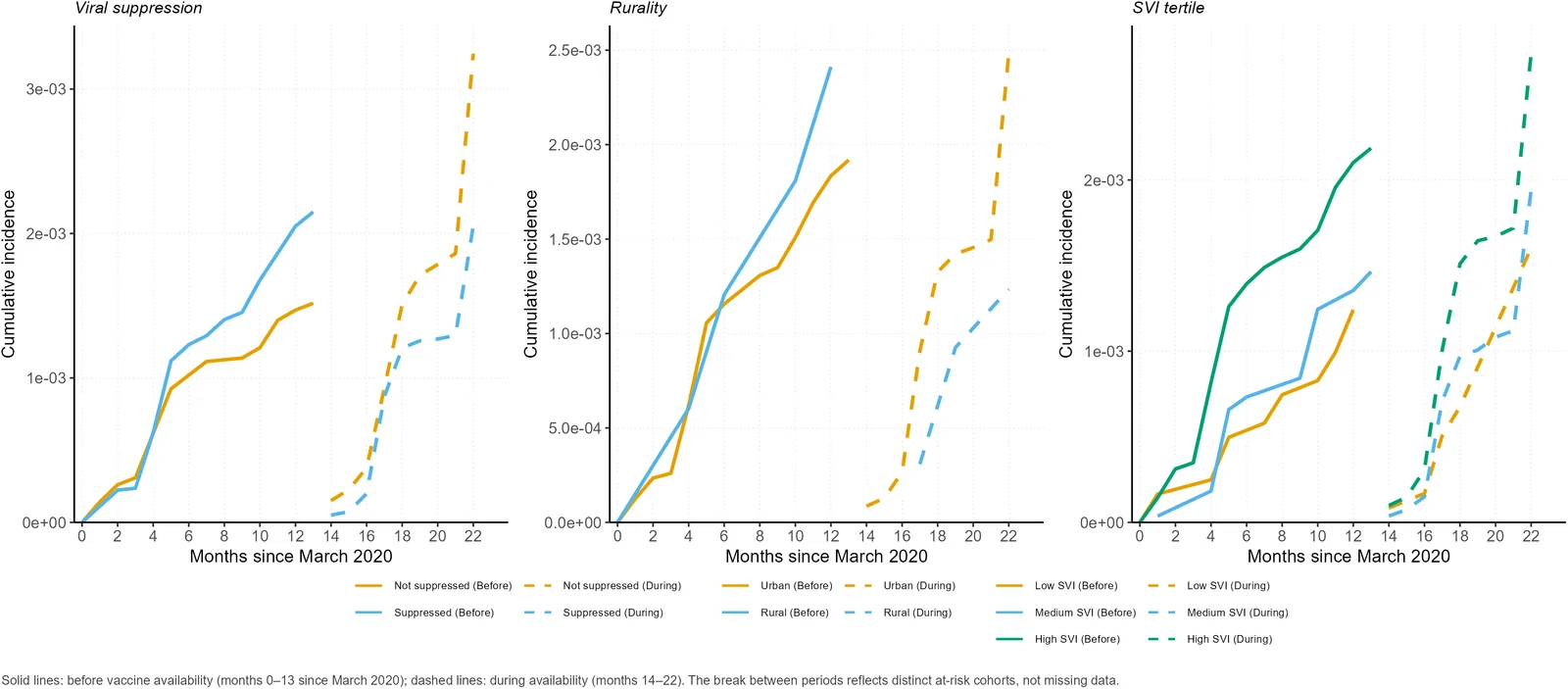
Cumulative incidence of COVID-19 deaths among people with HIV by viral suppression, rurality and social vulnerability index before and during vaccine availability, Florida, 2020–2021. Cumulative incidence of COVID-19 deaths among people with HIV by structural and community characteristics before and during COVID-19 vaccine availability, Florida, 2020–2021. Panels display viral suppression status, rural-urban residence classification, and Social Vulnerability Index (SVI) tertiles.

**Fig 3:**
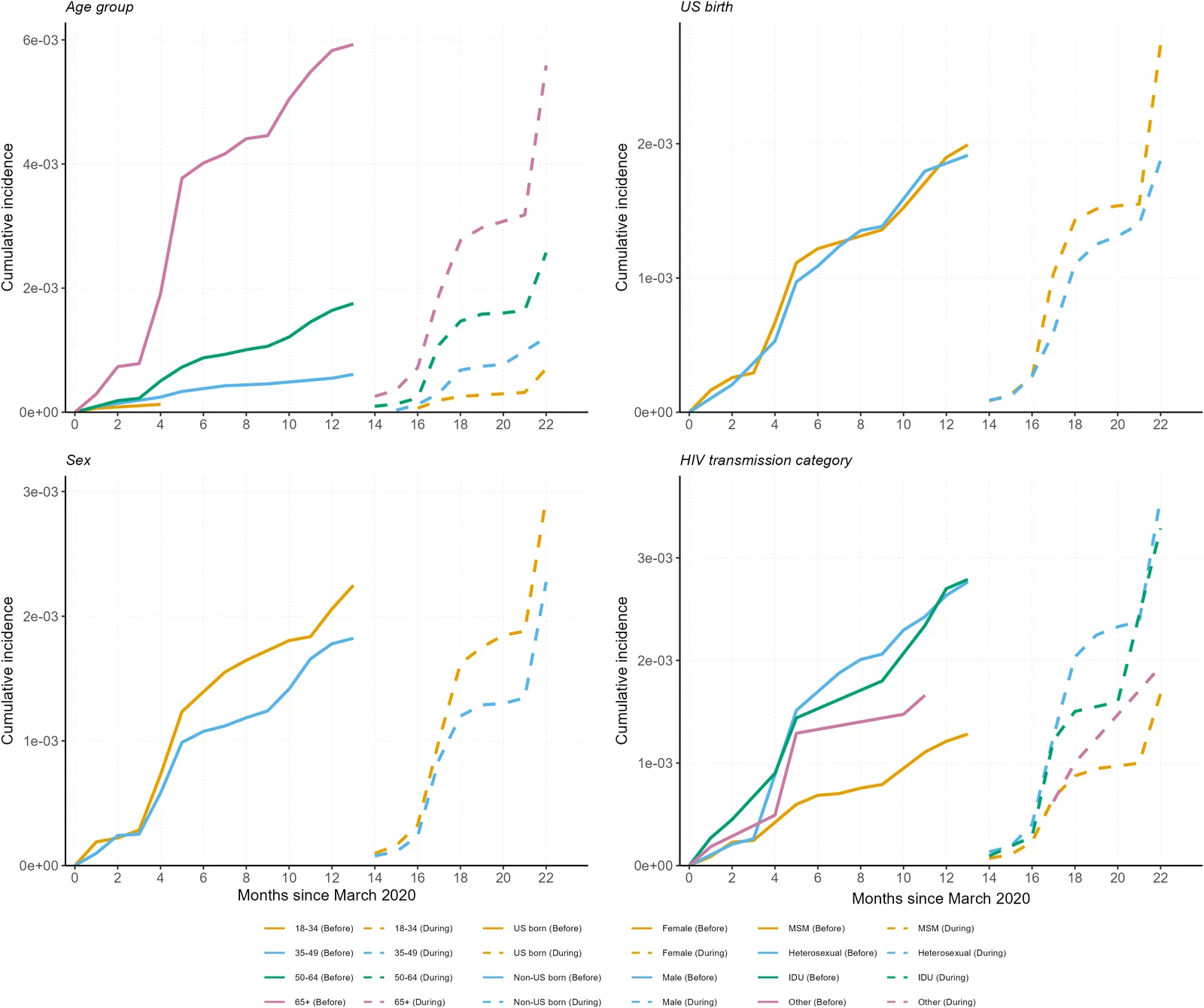
Cumulative incidence of COVID-19 deaths among people with HIV by age, US birth status, sex and transmission category before and during vaccine availability, Florida, 2020–2021. Cumulative incidence estimates are shown by individual characteristics. Panels display age group, sex, US birth status, and HIV transmission category. Solid lines represent the period before vaccine availability (March 1, 2020–April 30, 2021); dashed lines represent the period during vaccine availability (May 1, 2021–December 31, 2021).

Significant crude differences (*P* < 0.05) in the CIF for death curves were found across age, race/ethnicity, HIV transmission category, viral suppression, and SVI subgroups before and during vaccine availability. The CIF curves for birth sex were significant only during vaccine availability (*P* = 0.04). There were no significant differences by US-born status or rurality (Figs 1–3).

In the final proportional sub-distribution hazard model, the adjusted hazards for COVID-19 mortality relative to those aged 18–34 were higher for individuals aged 50–64 (during: HR 4.18, 95% CI 2.21–7.91; before: HR 5.43, 95% CI 2.68–11.03) and those ≥65 years (during: HR 9.13, 95% CI 4.78–17.41; before: HR 16.78, 95% CI 8.24–34.13) (Table 2).

**Table 2:** Adjusted sub-distribution hazard ratios of COVID-19 deaths among people with HIV before (March 1, 2020 – April 30, 2021) and during (May 1, 2021 – December 31, 2021) vaccine availability in Florida.

Characteristic	HR (95% CI) Before vaccine Model 1^c^	Type 3 P-value Model 1	HR (95% CI) During vaccine Model 1^c^	Type 3 P-value Model 1	HR (95% CI) Before vaccine Final model^d^	Type 3 P-value Final	HR (95% CI) During vaccine Final model^d^	Type 3 P-value Final
**Number of COVID-19 deaths**	350		291		350		291	
**N (person-time)**	119,168		116,787		119,168		116,787	
**Sex**		0.27		0.46		0.24		0.40
Male	Ref		Ref		Ref		Ref	
Female	0.86 (0.65–1.12)		0.89 (0.65–1.21)		0.85 (0.65–1.12)		0.88 (0.64–1.19)	
**Age at death (years)**		<.0001*		<.0001*		<.0001*		<.0001*
18–34	Ref		Ref		Ref		Ref	
35–49	1.89 (0.87–4.11)		1.76 (0.87–3.54)		1.89 (0.87–4.10)		1.76 (0.87–3.55)	
50–64	5.48 (2.70–11.13)		4.16 (2.20–7.87)		5.43 (2.68–11.03)		4.18 (2.21–7.91)	
65 and older	16.94 (8.32–34.48)		9.02 (4.73–17.22)		16.78 (8.24–34.13)		9.13 (4.78–17.41)	
**Race/ethnicity**		<.0001*		0.009*		<.0001*		0.02*
Non-Hispanic White	Ref		Ref		Ref		Ref	
Non-Hispanic Black	2.20 (1.56–3.10)		1.68 (1.19–2.38)		2.06 (1.45–2.91)		1.60 (1.13–2.27)	
Hispanic	2.31 (1.60–3.23)		1.04 (0.68–1.59)		2.24 (1.55–3.23)		1.03 (0.68–1.57)	
Other	1.80 (0.77–4.18)		1.02 (0.37–2.80)		1.74 (0.75–4.04)		0.99 (0.36–2.71)	
**HIV transmission category**		0.008*		0.003*		0.01*		0.004*
Men who have sex with men	Ref		Ref		Ref		Ref	
Injection drug use^a^	1.42 (0.98–2.05)		1.25 (0.83–1.88)		1.42 (0.98–2.05)		1.23 (0.82–1.85)	
Heterosexual	1.51 (1.09–2.09)		1.56 (1.08–2.24)		1.50 (1.09–2.08)		1.54 (1.07–2.21)	
Other	0.80 (0.45–1.40)		0.60 (0.31–1.16)		0.80 (0.46–1.42)		0.60 (0.31–1.17)	
**Born in US**		<.0005*		0.01*		<.0005*		0.02*
Yes	Ref		Ref		Ref		Ref	
No	0.61 (0.47–0.81)		0.66 (0.49–0.91)		0.60 (0.45–0.80)		0.68 (0.49–0.94)	
**Viral suppression** ^**b**^		0.56		<.0001*		0.60		<.0001*
Suppressed (Ref)	Ref		Ref		Ref		Ref	
Unsuppressed	1.07 (0.85–1.37)		1.79 (1.40–2.29)		1.07 (0.84–1.36)		1.79 (1.40–2.29)	
**Rural/urban status**	—	—	—	—		0.71		0.07
Urban					Ref		Ref	
Rural					0.89 (0.45–1.74)		0.40 (0.14–1.11)	
**Percent vaccinated by county**	—	—	—	—	0.88 (0.31–2.55)	0.81	0.46(0.16–1.38)	0.17
**Social Vulnerability Index (overall)**	—	—	—	—		0.13		0.07
Low					Ref		Ref	
Medium					1.17 (0.70–1.94)		1.43 (0.80–2.54)	
High					1.48 (0.93–2.37)		1.75 (1.04–2.97)	

HR= Adjusted Hazard Ratio; 95% CI= 95% Confidence Interval; *= significant <0.05 *P* value.

Ref = reference group; Type 3 *P* values indicate overall variable significance in the model.

Three COVID-19 deaths per period were excluded from the model due to missing covariate data; the final model included 350 events before and 291 events during vaccine availability.

^a^Injection Drug Use includes MSM and heterosexuals who are injection drug users.

^b^Viral suppression defined as HIV RNA < 200 copies/mL on most recent test (2020/2021).

^c^Model 1 = individual-level covariates only.

^d^Final Model = includes community-level factors.

During the vaccine period, elevated hazard of COVID-19 death relative to NHW was attenuated for Hispanics (during: HR 1.03, 95% CI 0.68–1.57; before: HR 2.24, 95% CI 1.55–3.23), but persisted for NHB (during: HR 1.60, 95% CI 1.13–2.27; before: HR 2.06, 95% CI 1.45–2.91) (Table 2). Regarding HIV transmission category, heterosexuals had higher hazard relative to MSM both before and during vaccine availability (during: HR 1.54, 95% CI 1.07–2.21; before: HR 1.50, 95% CI 1.09–2.08). Foreign-born individuals had lower hazard relative to US-born (during: HR 0.68, 95% CI 0.49–0.94; before: HR 0.60, 95% CI 0.45–0.80). Virally unsuppressed individuals had higher hazard relative to those suppressed during vaccine availability (during: HR 1.79, 95% CI 1.40–2.29; before: HR 1.07, 95% CI 0.84–1.36). During vaccine availability, PWH living in the highest tertile of overall social vulnerability had higher hazard compared with the lowest tertile (HR 1.75, 95% CI 1.04–2.97) (Table 2). No differences were noted by birth sex, rural-urban status, or county-level vaccination rates before or during vaccine availability (Table 2).

Crude (univariate) Fine–Gray estimates are provided for transparency in S1 Table. Sensitivity analyses shifting the vaccine-availability start date are shown in S2 Table (4-week) and S3 Table (8-week). Across the 4-week and 8-week definitions, estimates were directionally consistent with the primary 6-week analysis for age (HR 65 + : 9.32 for 4-week and 8.63 for 8-week, respectively), race/ethnicity (NHB remained elevated; Hispanic attenuation persisted), viral suppression (unsuppressed HR 1.76 for 4-week and HR 1.74 for 8-week, both p < 0.001), transmission category (heterosexual consistently elevated), foreign-born status (protective in both periods), and high social vulnerability (elevated during vaccine availability in both analyses). Event counts summed to 647 total COVID-19 deaths across all three definitions (343/304 for 4-week; 363/284 for 8-week), confirming internal consistency.

When analyzed by theme, significant differences emerged for Theme 1 during vaccine availability (Table 3). Compared to low SVI areas, mortality risk was elevated in highly socially vulnerable areas both during (HR 1.80, 95% CI 1.14–2.85) and before (HR 1.56, 95% CI 1.03–2.34) vaccine availability, and in moderately vulnerable areas it was only significant during vaccine availability (HR 1.67, 95% CI 1.02–2.72). Other SVI themes did not show significant differences. Sensitivity analyses shifting the vaccine-availability start date are shown in S2 Table (4-week) and S3 Table (8-week).

**Table 3:** Adjusted sub-distribution hazard ratios of COVID-19 deaths among people with HIV before (March 1, 2020 – April 30, 2021) and during (May 1, 2021 – December 31, 2021) vaccine availability in Florida, by SVI theme.

SVI theme	HR (95% CI) before vaccine	Type 3 P-value before	HR (95% CI) during vaccine	Type 3 P-value during
**Theme 1: Socioeconomic status disadvantage**		0.10		0.04*
Low	Ref	—	Ref	—
Medium	1.42 (0.92–2.19)		1.67 (1.02–2.72)	
High	1.56 (1.03–2.34)		1.80 (1.14–2.85)	
**Theme 2: Household composition and disability**		0.18		0.46
Low	Ref	—	Ref	—
Medium	1.17 (0.84–1.64)		1.03 (0.72–1.47)	
High	1.33 (0.97–1.81)		1.19 (0.86–1.65)	
**Theme 3: Minority status and language**		0.48		0.11
Low	Ref	—	Ref	—
Medium	0.90 (0.59–1.37)		0.91 (0.58–1.44)	
High	1.07 (0.69–1.66)		1.27 (0.81–2.01)	
**Theme 4: Housing type and transportation**		0.147		0.20
Low	Ref	—	Ref	—
Medium	0.99 (0.62–1.57)		1.27 (0.75–2.17)	
High	1.27 (0.82–1.97)		1.50 (0.90–2.49)	

HR = Adjusted Hazard Ratio; 95% CI = 95% Confidence Interval.

* = significant <0.05 *P* value; Ref = reference group.

Type 3 *P* values indicate overall variable significance in the model.

## Discussion

This retrospective study identified six key findings. First, overall COVID-19 mortality rates were higher during the vaccine availability period than before (27.5 vs. 22.3 deaths per 100,000 person-months), likely reflecting the Delta variant surge in mid-to-late 2021; however, subgroup patterns differed substantially from this overall trend. Second, relative to NHW, differences persisted for NHB while attenuation was observed for Hispanics during vaccine availability. Third, foreign-born individuals had lower hazard than US-born individuals in both periods. Fourth, higher COVID-19 mortality hazard during vaccine availability was observed among PWH without viral suppression relative to those with viral suppression. Fifth, heterosexuals consistently had higher hazards than MSM. Lastly, higher overall social vulnerability was associated with elevated hazard during vaccine availability, and Theme 1 (socioeconomic disadvantage) showed significant differences during vaccine availability.

While county-level studies in the general population have found that higher vaccination coverage was associated with lower overall COVID-19 mortality [13], our findings were more nuanced among PWH. During vaccine availability, the pre-vaccination disparity in COVID-19 mortality hazard between Hispanic and NHW PWH was attenuated, whereas the disparity between NHB and NHW PWH persisted. Because individual vaccination status was unavailable, these findings should not be interpreted as causal effects of vaccination uptake.

In our study, relative to NHWs, Hispanic PWH saw a significant reduction in COVID-19 mortality risk during vaccine availability, whilst NHB did not. In a Miami-Dade County Ryan White Program sample, lower vaccination rates among NHB PWH compared to Hispanic PWH were documented [23], potentially reflecting broader patterns of vaccine hesitancy and medical mistrust observed among Black communities [12,24]. Among PWH in Miami-Dade County, experiences of health care discrimination, structural barriers to vaccine access, and recommendations from HIV providers were not associated with complete vaccination; instead, full vaccination was associated with endorsing no COVID-19 vaccine misconceptions, receiving encouraging vaccine information, and perceiving that more than half of one’s social network was vaccinated [23]. Interventions tailored towards debunking misinformation, leveraging social networks, and enhancing community participation could increase vaccination uptake among NHB PWH, helping reduce differences in COVID-19 mortality.

Our study also found that foreign-born PWH had a lower risk of COVID-19 death compared to US-born PWH, a trend unchanged by vaccine availability. Foreign-born immigrants, particularly Latinos, usually have lower all-cause mortality rates than US-born non-Hispanic White populations, a phenomenon termed the “Latino paradox” [25–27]. Factors contributing to this advantage include selective migration of healthier individuals, healthier lifestyle practices, and higher vaccination rates. In a national analysis conducted from April 22, 2021, through January 29, 2022, 80.9% of foreign-born adults reported receiving at least one COVID-19 vaccine dose, compared with 72.6% of U.S.-born adults, while 6.0% of foreign-born adults were reluctant to be vaccinated, compared with 15.8% of U.S.-born adults [28].

In our study, virally unsuppressed PWH had higher COVID-19 mortality hazard during vaccine availability compared with those suppressed. Viral suppression may also reflect engagement in HIV care; in New York City, individuals retained in care maintained high viral suppression rates during COVID-19 [29]. In New York State, completed COVID-19 vaccination was also more common among PWH receiving HIV care than among those without surveillance evidence of care (69.2% vs. 29.1%) and among those virally suppressed versus unsuppressed (72.0% vs. 38.1%) [15]. Because we did not measure individual vaccine uptake, the associations in our study should be interpreted as period comparisons defined by vaccine availability rather than vaccination receipt.

Among injection drug use transmission category, COVID-19 mortality risk decreased with vaccine availability, while it remained unchanged for heterosexuals compared to MSM. This reduction might be due to increased engagement with healthcare services during the pandemic, though this hypothesis requires further investigation.

During both time periods, PWH in high social vulnerability areas, as defined by the socioeconomic theme, faced greater mortality risk than those in low vulnerability areas. Similar patterns have been observed in the general population, where socioeconomic disadvantage is associated with higher COVID-19 mortality, likely through multiple pathways including reduced healthcare access and greater occupational exposure [7,8].

Unlike a pooled descriptive analysis in PWH which showed that the case-fatality rate among males was nearly twice that among females [6], we did not observe sex differences in COVID-19 mortality among PWH. This may be explained by the equal access to HIV-specific healthcare services across gender which may help mitigate general healthcare access differences based on sex. Additionally, PWH may have higher health literacy regarding infection risks and prevention behaviors, which could reduce differences in outcomes between males and females or between rural and urban residents.

### Limitations

This study has limitations. First, this study relied on HIV surveillance records that do not include information on individual-level COVID-19 vaccination. Despite this, we were able to leverage ZIP Code information to link public data sources to estimate vaccine coverage at the county level and community social vulnerability at the ZCTA level.

Second, the sub-distribution hazard model limits effect size interpretation to direction (increase or decrease) rather than risk magnitude, though comparisons within the same model remain valid [30]. Therefore, interpretations can still be made in terms of differences before and during for variables within the same model. Thirdly, unmeasured confounding factors, such as individual socioeconomic status, underlying health conditions, or access to healthcare services, could affect the results, and should be addressed in future studies. Future studies could use more accurate, timely data sources to address this. Finally, the study’s findings, based on data up to December 2021, miss the effects of later vaccination efforts and emerging variants.

Nonetheless, this study provides valuable insights into vaccination differences and underscores the need for tailored public health interventions. Furthermore, this research provides an expanded understanding of differences by examining both individual- and community-level factors, enabling the development of more effective solutions which account for the dynamic interaction between community and individual health determinants.

### Public health implications

The findings of this study offer several important public health implications. Ensuring timely access to vaccines for PWH is crucial, especially for older PWH given their increased risk of mortality. Tailored vaccination campaigns are needed in communities with lower vaccination coverage, including counties with higher social vulnerability [31]. Improving access to healthcare services among NHB is essential to reduce persistent differences in health outcomes. Programs to build trust in the healthcare system among minority groups should address historical and ongoing healthcare discrimination and mistrust. Launching educational campaigns to combat misinformation and vaccine hesitancy, with an emphasis on the safety and efficacy of vaccines, is vital. Leveraging social networks and community leaders to encourage vaccination, particularly among PWH and other vulnerable populations, can also enhance vaccination rates.

Developing culturally sensitive health communication strategies ensures vaccine information is accessible and relevant across diverse populations. Ongoing engagement with healthcare services for PWH supports continuous care and timely vaccinations. Establishing systems to track vaccination status and outcomes for PWH and other vulnerable populations, not only for COVID-19, can identify service gaps and guide targeted interventions. Allocating resources to high-social-vulnerability areas ensures adequate infrastructure for vaccine distribution and healthcare delivery, while advocating for policies that address systemic barriers promotes health equity. Assessing community barriers and misinformation through surveys, especially in partnership with community-based organizations, supports well-tailored public health efforts and addresses medical mistrust. Addressing these areas can help public health initiatives reduce differences, improve health equity, and strengthen vaccination campaigns and healthcare delivery.

## Supporting information

S1 Table Univariate crude sub-distribution hazard ratios for COVID-19 mortality among people with HIV in Florida, before and during vaccine availability, 2020–2021. (DOCX)

S2 Table Sensitivity analysis (4-week vaccine-availability start): Adjusted sub-distribution hazard ratios of COVID-19 deaths among people with HIV, before (March 1, 2020 – March 31, 2021) and during (April 1, 2021 – December 31, 2021) vaccine availability in Florida. (DOCX)

S3 Table Sensitivity analysis (8-week vaccine-availability start): Adjusted sub-distribution hazard ratios of COVID-19 deaths among people with HIV, before (March 1, 2020 – May 31, 2021) and during (June 1, 2021 – December 31, 2021) vaccine availability in Florida. (DOCX)
