# Supplementary material for: COVID-19 mortality risk among people with HIV in Florida before and after the introduction of COVID-19 vaccines: A population-based study
Source: PLoS One. 2026 Sep 18;21(9):e0358543. doi: 10.1371/journal.pone.0358543 (PMC13588381; doi:10.1371/journal.pone.0358543)
Supplement: Supplementary file 1 [file pone.0358543.s001.docx]

**S1 Table. Univariate crude sub-distribution hazard ratios for COVID-19 mortality among people with HIV in Florida, before and during vaccine availability, 2020–2021.**

| **Variable** | **Level** | **Before vaccine availability sHR (95% CI)^a,b^** | **p-value^c^** | **During vaccine availability sHR (95% CI)^a,b^** | **p-value^c^** |
| --- | --- | --- | --- | --- | --- |
| Age, years | 18–34 | Ref.^d^ | 1.00 | Ref | 1.00 |
| Age, years | 35–49 | 1.90 (0.87–4.14) | 0.10 | 1.77 (0.88–3.58) | 0.11 |
| Age, years | 50–64 | 5.23 (2.56–10.67) | <0.001* | 3.95 (2.07–7.51) | <0.001* |
| Age, years | ≥65 | 16.08 (7.90–32.72) | <0.001* | 8.73 (4.56–16.73) | <0.001* |
| Sex | Male | Ref.^d^ | 1.00 | Ref.d | 1.00 |
| Sex | Female | 1.24 (0.98–1.57) | 0.07 | 1.32 (1.03–1.71) | 0.03* |
| Race/ethnicity | Non-Hispanic White | Ref | 1.00 | Ref | 1.00 |
| Race/ethnicity | Hispanic | 1.48 (1.06–2.08) | 0.02* | 0.76 (0.52–1.13) | 0.17 |
| Race/ethnicity | Non-Hispanic Black | 1.99 (1.48–2.68) | <0.001* | 1.71 (1.27–2.29) | 0.0004* |
| Race/ethnicity | Other | 1.35 (0.58–3.13) | 0.49 | 0.85 (0.31–2.34) | 0.76 |
| US-born | Yes | Ref | 1.00 | Ref | 1.00 |
| US-born | No | 0.81 (0.63–1.04) | 0.10 | 0.75 (0.57–1.00) | 0.047* |
| Transmission category | MSM | Ref | 1.00 | Ref | 1.00 |
| Transmission category | IDU^f^ | 2.32 (1.66–3.25) | <0.001* | 1.98 (1.34–2.93) | <0.0006* |
| Transmission category | Heterosexual | 2.06 (1.61–2.64) | <0.001* | 2.15 (1.65–2.80) | <0.001* |
| Transmission category | Other | 1.12 (0.67–1.87) | 0.658 | 0.93 (0.51–1.70) | 0.82 |
| Viral suppression^g^ | Suppressed | Ref.d | 1.00 | Ref.d | 1.00 |
| Viral suppression^g^ | Unsuppressed | 1.02 (0.81–1.28) | 0.90 | 1.68 (1.32–2.14) | <0.001* |
| Rural/urban | Urban | Ref.d | 1.00 | Ref.d | 1.00 |
| Rural/urban | Rural | 1.04 (0.54–2.02) | 0.90 | 0.55 (0.20–1.47) | 0.23 |
| SVI theme (overall)^h^ | Low | Ref.d | 1.00 | Ref.d | 1.00 |
| SVI theme (overall)^h^ | Medium | 1.24 (0.74–2.06) | 0.418 | 1.47 (0.83–2.62) | 0.191 |
| SVI theme (overall)^h^ | High | 1.79 (1.14–2.83) | 0.012* | 1.99 (1.18–3.36) | 0.010* |

Note: aHR = Adjusted Hazard Ratio; b95% CI = 95% Confidence Interval; c* = significant <0.05 P value; dRef = reference group; eType 3 P values indicate overall variable significance in the model; fInjection Drug Use includes MSM and heterosexuals who are injection drug users; gViral suppression defined as HIV RNA <200 copies/mL on most recent test (2020/2021); hSVI tertiles: Low (0–0.333), Medium (0.334–0.666), High (0.667–1); iModel 1 = individual-level covariates only. Final Model = includes community-level factors.
