# Supplementary material for: COVID-19 mortality risk among people with HIV in Florida before and after the introduction of COVID-19 vaccines: A population-based study
Source: PLoS One. 2026 Sep 18;21(9):e0358543. doi: 10.1371/journal.pone.0358543 (PMC13588381; doi:10.1371/journal.pone.0358543)
Supplement: Supplementary file 2 [file pone.0358543.s002.docx]

**S2 Table. Sensitivity analysis (4-week vaccine-availability start): Adjusted sub-distribution hazard ratios of COVID-19 deaths among people with HIV, before (March 1, 2020 – March 31, 2021) and during (April 1, 2021 – December 31, 2021) vaccine availability in Florida.**

| **Characteristic** | **HR (95% CI) Before Model 1** | **Type 3 P** | **HR (95% CI) Before Final** | **Type 3 P** | **HR (95% CI) During Model 1** | **Type 3 P** | **HR (95% CI) During Final** | **Type 3 P** |
| --- | --- | --- | --- | --- | --- | --- | --- | --- |
| **COVID-19 deaths** | 340 |  | 340 |  | 301 |  | 301 |  |
| N | 119,316 |  | 119,316 |  | 116,963 |  | 116,963 |  |
| **Sex** |  | 0.13 |  | 0.12 |  | 0.74 |  | 0.66 |
| Male | Ref |  | Ref |  | Ref |  | Ref |  |
| Female | 0.81 (0.61–1.07) |  | 0.80 (0.61–1.06) |  | 0.95 (0.70–1.29) |  | 0.93 (0.69–1.27) |  |
| **Age (years)** |  | <.0001* |  | <.0001* |  | <.0001* |  | <.0001* |
| 18–34 | Ref |  | Ref |  | Ref |  | Ref |  |
| 35–49 | 1.78 (0.81–3.88) |  | 1.77 (0.81–3.86) |  | 1.85 (0.92–3.71) |  | 1.85 (0.92–3.72) |  |
| 50–64 | 5.24 (2.58–10.66) |  | 5.20 (2.56–10.58) |  | 4.37 (2.31–8.24) |  | 4.38 (2.32–8.27) |  |
| 65 and older | 16.67 (8.19–33.93) |  | 16.56 (8.13–33.71) |  | 9.26 (4.86–17.65) |  | 9.32 (4.89–17.76) |  |
| **Race/ethnicity** |  | <.0001* |  | <.0001* |  | 0.008* |  | 0.02* |
| Non-Hispanic White | Ref |  | Ref |  | Ref |  | Ref |  |
| Non-Hispanic Black | 2.21 (1.56–3.13) |  | 2.07 (1.45–2.95) |  | 1.69 (1.20–2.37) |  | 1.60 (1.14–2.25) |  |
| Hispanic | 2.30 (1.58–3.33) |  | 2.25 (1.54–3.27) |  | 1.09 (0.72–1.64) |  | 1.06 (0.71–1.60) |  |
| Other | 1.86 (0.80–4.35) |  | 1.81 (0.78–4.21) |  | 0.99 (0.36–2.71) |  | 0.95 (0.35–2.62) |  |
| **Transmission category** |  | 0.007* |  | 0.009* |  | 0.004* |  | 0.004* |
| MSM | Ref |  | Ref |  | Ref |  | Ref |  |
| IDU | 1.46 (1.00–2.12) |  | 1.45 (1.00–2.10) |  | 1.21 (0.81–1.81) |  | 1.20 (0.80–1.79) |  |
| Heterosexual | 1.56 (1.12–2.17) |  | 1.54 (1.11–2.15) |  | 1.50 (1.05–2.14) |  | 1.50 (1.04–2.13) |  |
| Other | 0.83 (0.47–1.47) |  | 0.84 (0.47–1.48) |  | 0.57 (0.30–1.10) |  | 0.57 (0.30–1.11) |  |
| **Born in US** |  | 0.0007* |  | <.001* |  | 0.007* |  | 0.01* |
| Yes | Ref |  | Ref |  | Ref |  | Ref |  |
| No | 0.62 (0.47–0.81) |  | 0.61 (0.46–0.81) |  | 0.66 (0.49–0.89) |  | 0.66 (0.48–0.92) |  |
| **Viral suppression** |  | 0.50 |  | 0.52 |  | <.0001* |  | <.0001* |
| Suppressed | Ref |  | Ref |  | Ref |  | Ref |  |
| Unsuppressed | 1.09 (0.85–1.39) |  | 1.08 (0.85–1.39) |  | 1.76 (1.38–2.24) |  | 1.76 (1.38–2.24) |  |
| **Rural/urban** | — | — |  | 0.73 | — | — |  | 0.07 |
| Urban | — | — | Ref |  | — | — | Ref |  |
| **Rural** | — | — | 0.88 (0.44–1.74) |  | — | — | 0.40 (0.15–1.08) |  |
| **SVI overall** | — | — |  | 0.11 | — | — |  | 0.073 |
| Low | — | — | Ref |  | — | — | Ref |  |
| Medium | — | — | 1.10 (0.66–1.84) |  | — | — | 1.51 (0.86–2.68) |  |
| High | — | — | 1.45 (0.91–2.33) |  | — | — | 1.80 (1.06–3.04) |  |
| **Percent vaccinated** | — | — | 0.74 (0.25–2.17) | 0.61 | — | — | 0.57 (0.19–1.69) | 0.31 |

*Note*: ^a^HR= Adjusted Hazard Ratio; ^b^95% CI= 95% Confidence Interval; ^c^*= significant <0.05 *P* value

^d^Ref = reference group; ^e^Type 3 *P* values indicate overall variable significance in the model.

^f^Injection Drug Use includes MSM and heterosexuals who are injection drug users.

^g^ Viral suppression defined as HIV RNA <200 copies/mL on most recent test (2020/2021).

^h^SVI tertiles: Low (0–0.333), Medium (0.334–0.666), High (0.667–1).

^i^Model 1 = individual-level covariates only. Final Model = includes community-level factors
