# Supplementary material for: COVID-19 mortality risk among people with HIV in Florida before and after the introduction of COVID-19 vaccines: A population-based study
Source: PLoS One. 2026 Sep 18;21(9):e0358543. doi: 10.1371/journal.pone.0358543 (PMC13588381; doi:10.1371/journal.pone.0358543)
Supplement: Supplementary file 3 [file pone.0358543.s003.docx]

**S3 Table. Sensitivity analysis (8-week vaccine-availability start): Adjusted sub-distribution hazard ratios of COVID-19 deaths among people with HIV, before (March 1, 2020 – May 31, 2021) and during (June 1, 2021 – December 31, 2021) vaccine availability in Florida.**

| **Characteristic** | **HR (95% CI) Before Model 1** | **Type 3 P** | **HR (95% CI) Before Final** | **Type 3 P** | **HR (95% CI) During Model 1** | **Type 3 P** | **HR (95% CI) During Final** | **Type 3 P** |
| --- | --- | --- | --- | --- | --- | --- | --- | --- |
| **COVID-19 deaths** | 360 |  | 360 |  | 281 |  | 281 |  |
| N | 119,316 |  | 119,316 |  | 116,595 |  | 116,595 |  |
| **Sex** |  | 0.27 |  | 0.25 |  | 0.45 |  | 0.39 |
| Male | Ref |  | Ref |  | Ref |  | Ref |  |
| Female | 0.86 (0.66–1.13) |  | 0.85 (0.65–1.12) |  | 0.89 (0.65–1.21) |  | 0.87 (0.64–1.19) |  |
| **Age (years)** |  | <.0001* |  | <.0001* |  | <.0001* |  | <.0001* |
| 18–34 | Ref |  | Ref |  | Ref |  | Ref |  |
| 35–49 | 1.90 (0.87–4.12) |  | 1.89 (0.87–4.10) |  | 1.75 (0.87–3.53) |  | 1.75 (0.87–3.52) |  |
| 50–64 | 5.70 (2.81–11.57) |  | 5.64 (2.78–11.45) |  | 3.97 (2.10–7.51) |  | 4.00 (2.11–7.56) |  |
| 65 and older | 17.57 (8.64–35.72) |  | 17.36 (8.54–35.30) |  | 8.51 (4.45–16.26) |  | 8.63 (4.51–16.48) |  |
| **Race/ethnicity** |  | <.0001* |  | <.0001* |  | 0.012* |  | 0.031* |
| Non-Hispanic White | Ref |  | Ref |  | Ref |  | Ref |  |
| Non-Hispanic Black | 2.20 (1.57–3.08) |  | 2.06 (1.47–2.90) |  | 1.67 (1.17–2.37) |  | 1.58 (1.11–2.26) |  |
| Hispanic | 2.28 (1.59–3.28) |  | 2.21 (1.54–3.17) |  | 1.02 (0.67–1.57) |  | 1.02 (0.66–1.56) |  |
| Other | 1.74 (0.75–4.05) |  | 1.69 (0.73–3.91) |  | 1.05 (0.38–2.88) |  | 1.01 (0.37–2.79) |  |
| **Transmission category** |  | 0.007* |  | 0.008* |  | 0.004* |  | 0.005* |
| MSM | Ref |  | Ref |  | Ref |  | Ref |  |
| IDU | 1.39 (0.97–2.00) |  | 1.39 (0.97–2.00) |  | 1.29 (0.85–1.95) |  | 1.26 (0.83–1.92) |  |
| Heterosexual | 1.49 (1.08–2.06) |  | 1.49 (1.08–2.05) |  | 1.58 (1.10–2.29) |  | 1.57 (1.08–2.26) |  |
| Other | 0.76 (0.43–1.33) |  | 0.77 (0.44–1.35) |  | 0.64 (0.33–1.24) |  | 0.64 (0.33–1.25) |  |
| **Born in US** |  | 0.0005* |  | 0.0005* |  | 0.010* |  | 0.02* |
| Yes | Ref |  | Ref |  | Ref |  | Ref |  |
| No | 0.62 (0.47–0.81) |  | 0.61 (0.46–0.80) |  | 0.66 (0.48–0.91) |  | 0.68 (0.49–0.94) |  |
| **Viral suppression** |  | 0.49 |  | 0.53 |  | <.0001* |  | <.0001* |
| Suppressed | Ref |  | Ref |  | Ref |  | Ref |  |
| Unsuppressed | 1.09 (0.86–1.38) |  | 1.08 (0.85–1.37) |  | 1.74 (1.35–2.23) |  | 1.74 (1.36–2.24) |  |
| **Rural/urban** | — | — |  | 0.67 | — | — |  | 0.08 |
| Urban | — | — | Ref |  | — | — | Ref |  |
| **Rural** | — | — | 0.87 (0.44–1.70) |  | — | — | 0.41 (0.15–1.10) |  |
| **SVI overall** | — | — |  | 0.13 | — | — |  | 0.069 |
| Low | — | — | Ref |  | — | — | Ref |  |
| Medium | — | — | 1.13 (0.69–1.86) |  | — | — | 1.50 (0.83–2.71) |  |
| High | — | — | 1.45 (0.92–2.29) |  | — | — | 1.83 (1.06–3.16) |  |
| **Percent vaccinated** | — | — | 0.93 (0.33–2.65) | 0.90 | — | — | 0.42 (0.14–1.29) | 0.13 |

*Note*: ^a^HR= Adjusted Hazard Ratio; ^b^95% CI= 95% Confidence Interval; ^c^*= significant <0.05 *P* value

^d^Ref = reference group; ^e^Type 3 *P* values indicate overall variable significance in the model.

^f^Injection Drug Use includes MSM and heterosexuals who are injection drug users.

^g^ Viral suppression defined as HIV RNA <200 copies/mL on most recent test (2020/2021).

^h^SVI tertiles: Low (0–0.333), Medium (0.334–0.666), High (0.667–1).

^i^Model 1 = individual-level covariates only. Final Model = includes community-level factors
